# Surfing the web during pandemic flu: availability of World Health Organization recommendations on prevention

**DOI:** 10.1186/1471-2458-10-561

**Published:** 2010-09-20

**Authors:** Francesco Gesualdo, Mariateresa Romano, Elisabetta Pandolfi, Caterina Rizzo, Lucilla Ravà, Daniela Lucente, Alberto E Tozzi

**Affiliations:** 1 Ospedale Pediatrico Bambino Gesù, Piazza Sant'Onofrio 4, Rome, Italy; 2 Istituto Superiore di Sanità, Viale Regina Elena 299, Rome, Italy

## Abstract

**Background:**

People often search for information on influenza A(H1N1)v prevention on the web. The extent to which information found on the Internet is consistent with recommendations issued by the World Health Organization is unknown.

**Methods:**

We conducted a search for "swine flu" accessing 3 of the most popular search engines through different proxy servers located in 4 English-speaking countries (Australia, Canada, UK, USA). We explored each site resulting from the searches, up to 4 clicks starting from the search engine page, analyzing availability of World Health Organization recommendations for swine flu prevention.

**Results:**

Information on hand cleaning was reported on 79% of the 147 websites analyzed; staying home when sick was reported on 77.5% of the websites; disposing tissues after sneezing on 75.5% of the websites. Availability of other recommendations was lower. The probability of finding preventative recommendations consistent with World Health Organization varied by country, type of website, and search engine.

**Conclusions:**

Despite media coverage on H1N1 influenza, relevant information for prevention is not easily found on the web. Strategies to improve information delivery to the general public through this channel should be improved.

## Background

In June 2009 the World Health Organization's director-general Dr. Margaret Chan, raising the pandemic alert level to phase six, officially declared the first flu pandemic of the 21st century [1].

According with WHO recommendations, during a pandemic the adoption of non-pharmaceutical preventive measures represents one of the most important interventions in mitigating the spread of the infection in the community [2,3]. Of such preventive measures, some are supported by scientific studies (hand washing, isolation of the sick, use of protective equipment), others are recommended by groups of experts (respiratory etiquette [2]).

However, in order to be effective, non-pharmaceutical preventive measures must gain community acceptance [3]. Thus, in order to achieve a change of behavior in the general public and favor the implementation of such recommendations, health professionals, using communication strategies, have informed the population through different channels, including mass-reach broadcast media advertising (television and radio advertisements or programs), print-based materials, and audiovisual materials [4].

The role of the Internet as a platform for delivering public health interventions to specific patient groups and to the general public is constantly increasing, due in particular to its disseminating potential [5]: the worldwide penetration of the Internet is increasing and the use of this medium for seeking health information is frequent [4]. Moreover, the Internet potential for individual tailoring and interactivity is superior to that of other high reach-delivery channels [5].

Integration of the Internet with the more classical media as well as with the novel, alternative formats (email, interactive digital TV, SMS texts, podcasts) for social marketing can allow to reach specific groups whose characteristics are different from those of the general population [6].

In the evolving context of the swine flu pandemic the World Health Organization (WHO) issued specific recommendations for individuals, addressing a set of behavior changes that can help prevent the spread of the influenza virus. These recommendations are accessible on the WHO website [7].

Recommendations for H1N1 flu prevention may not be available or easily accessible on the Internet. Chances to get appropriate information about swine flu might also vary with the different geographic areas from which the information is sought.

The objective of our research is to analyze the probability that an average Internet user has to get appropriate information about H1N1 flu prevention in four different English-speaking countries when searching advice through generic web search engines.

## Methods

### Study design

We simulated a search for generic information on pandemic flu in four English speaking countries using generic web search engines, and analyzed the contents of the first page hits for presence of recommendations as reported by WHO [7]. Selected countries included Australia, Canada, UK, and USA.

### Search string and search strategy

We identified the most used search strings for seeking generic information on influenza A(H1N1)v on the web through "Google Trends", an online application that shows how often a particular search-term is entered relative to the total search-volume across different regions of the world [8]. We compared three different search strings ("influenza", "swine flu", "H1N1 flu"), and verified that *swine flu *had been the most frequently used search string in the four countries considered in our study during the 30 days prior to the investigation.

On August 13^th ^2009 we performed multiple searches for "swine flu" using the three most popular search engines (Google, Yahoo, MSN-Bing). The search engines were accessed through four different proxy servers, located in each of the 4 above mentioned countries. Through this strategy, we obtained results that reflected exactly what would be obtained launching the search from the four different countries.

In order to review the web pages as they were found on the date of the search we downloaded all resulting web pages with an off line browser and stored them in CD-ROM supports which were used for the review process.

### Review process

We analyzed all the Internet pages shown in the first page of the search results.

For each webpage resulting from the searches, we recorded the country where the search was launched from, and the search engine we obtained it from. In the revision process two of the authors reviewed all the web pages content.

We recorded the type of web site (Public Health Agency, university/hospital/research, news, drug company/commercial, other types of website including Wikipedia and blogs), and explored each Internet page and all its links up to 4 clicks starting from the search engine webpage. In order to compare website contents we selected all the recommendations for swine flu prevention listed in the WHO recommendation webpage [7], and created a checklist of 21 items, each item being relevant to one of the recommendations (Table 1). Through this checklist we verified whether each specific prevention item was available on the reviewed Internet pages. We defined an item as available when the information was reached and consistent with the WHO recommendation.

**Table 1:** Frequency and percentage of websites reporting recommendations consistent with WHO (total number of websites: 147 [13])

Recommendation	no. (%)
**Prevention measures**	
avoid touching mouth and nose	98 (66.67%)
hand cleaning	116 (78.91%)
avoid close contact with sick people	91 (61.90%)
reduce time spent in crowds	86 (59.86%)
improve airflow in living place	30 (20.41%)
practice good health habits	43 (29.25%)
	
**Mask wearing indications**	
if you are not sick: mask not needed	82 (59.86%)
caregivers: mask needed when in close contact	76 (51.70%)
if you are sick: mask needed when in close contact/travel	74 (50.34%)
	
**If you have the illness**	
stay at home	114 (77.55%)
take rest and drink fluids	67 (45.58%)
avoid contact with other people	92 (62.59%)
cover mouth-nose with a tissue when coughing-sneezing	110 (74.83%)
dispose your tissue after using it	111 (75.51%)
if you do not have a tissue, cover mouth-nose with crook of elbow	46 (34.01%)
clean your hands after sneezing	96 (65.31%)
inform family and friend about your illness	26 (17.69%)
contact a health professional before travelling to health facility	47 (31.97%)
do not stop breastfeeding	61 (41.50%)
	
**Eating pork is safe**	88 (59.86%)
	
**No travelling restriction related to pandemic**	71 (49.66%)

### Statistical analysis

We calculated the frequency of available items included in the WHO recommendation list by type of web site, country, and search engine, within 4 clicks. Since different search engines may report overlapping results, we calculated proportions excluding multiple overlapping results in the analysis by country. To compare the obtained figures we used the Chi square test. We then performed a logistic regression analysis with the dependent variable being the availability of specific recommendations and the independent variables being country, search engine and type of web site. Associations at the multivariate analysis were expressed with adjusted odds ratios and 95% confidence intervals. All statistical analyses were considered significant at the 0.05 level. We used Stata version 10.0 for the analysis.

## Results

We found a total of 147 websites, resulting from the search in the four English-speaking countries with the three web search engines: Australia (30 sites), Canada (40 sites), US (40 sites), UK (37 sites). For a complete list of the URLs resulting from the searches see Additional file 1: URL list.

Most of the websites were from Public Health Agencies and from news providers: these two categories accounted for a proportion of 37% and 40% respectively. In fewer cases the websites were from Universities, Hospitals, Research Institutes, or blogs. Few websites were commercial or from drug industries.

The first hit reported in each search result page was always a website from a Public Health Agency, with the exception of MSN searches from Canada (first hit: news provider) and US (first hit: Wikipedia)

Overall, the information availability differed between each recommendation. We found that recommendations about hand cleaning, staying home when sick, use of tissue when coughing or sneezing and tissue disposal were the most frequently shown. One step of the respiratory etiquette, covering mouth and nose with the sleeve or with the elbow crook when coughing or sneezing, was among the less represented information on the web. Other information rarely reported included airflow improvement, keeping good health habits, informing family and friends when sick, contacting health professionals before travelling to health facilities (Table 1).

In the univariate analysis on availability of recommendations by country, we found that information about resting and drinking fluids when sick, breastfeeding despite illness, no restriction to travel to and from any country during pandemic, and contacting health professionals before travelling to health facilities were more represented in searches conducted from the United Kingdom than in searches conducted from the other countries. Presence of information varied significantly among the four countries also regarding communication to family and friends when sick, and one of the mask-wearing indications.

We performed then a logistic regression model to explore the independent effect of country adjusting for type of website and search engine. The reference country used for this analysis was UK. (Table 2)

**Table 2:** Adjusted ORs and 95% CI of finding single recommendations during web search by country (multivariable analysis, reference country: UK)

Prevention measures	Australia		Canada		USA	
			
	**OR**^a^ (95% CI^b^)	P	**OR**^a^ (95% CI^b^)	p	**OR**^a^ (95% CI^b^)	p
avoid touching mouth and nose	1.058 (0.361 - 3.101)	0.918	1.438 (0.512 - 4.039)	0.490	1.613 (0.536 - 4.854)	0.395
hand cleaning	0.128 (0.025 - 0.655)	0.014	0.322 (0.075 - 1.385)	0.128	0.504 (0.107 - 2.380)	0.387
avoid close contact with sick people	1.587 (0.540 - 4.663)	0.401	0.777 (0.291 - 2.075)	0.615	1.859 (0.643 - 5.377)	0.253
reduce time spent in crowds	0.530 (0.179 - 1.575)	0.254	1.453 (0.517 - 4.085)	0.479	2.082 (0.686 - 6.321)	0.196
improve airflow in living place	0.463 (0.124 - 1.721)	0.250	0.529 (0.158 - 1.764)	0.300	0.644 (0.187 - 2.215)	0.485
practice good health habits	0.908 (0.302 - 2.727)	0.863	0.850 (0.294 - 2.459)	0.764	0.697 (0.227 - 2.142)	0.529
if you are not sick: mask not needed	0.213 (0.067 - 0.684)	0.009	0.362 (0.124 - 1.058)	0.063	0.630 (0.201 - 1.970)	0.427
caregivers: mask when in close contact	0.704 (0.246 - 2.015)	0.513	0.846 (0.312 - 2.297)	0.743	2.285 (0.792 - 6.589)	0.126
sick: mask when close contact/travel	1.431 (0.523 - 3.916)	0.485	1.923 (0.737 - 5.019)	0.181	3.882 (1.403 - 10.743)	0.009
stay at home	0.141 (0.027 - 0.723)	0.019	0.411 (0.097 - 1.739)	0.227	0.577 (0.126 - 2.642)	0.479
take rest and drink fluids	0.136 (0.044 - 0.417)	0.000	0.262 (0.094 - 0.730)	0.010	0.242 (0.083 - 0.708)	0.010
avoid contact with other people	1.318 (0.414 - 4.199)	0.640	1.886 (0.632 - 5.632)	0.255	2.834 (0.863 - 9.301)	0.086
use tissue when coughing-sneezing	0.296 (0.075 - 1.165)	0.082	0.433 (0.123 - 1.533)	0.195	0.429 (0.112 - 1.635)	0.215
dispose your tissue after using it	0.220 (0.052 - 0.934)	0.040	0.338 (0.090 - 1.274)	0.109	0.332 (0.082 - 1.346)	0.122
sleeve - crook of elbow	1.018 (0.335 - 3.091)	0.974	3.943 (1.363 - 11.405)	0.011	1.357 (0.444 - 4.149)	0.592
clean your hands after sneezing	0.253 (0.076 - 0.837)	0.024	0.998 (0.326 - 3.058)	0.997	1.188 (0.357 - 3.950)	0.779
inform family and friend about your illness	0.478 (0.143 - 1.596)	0.230	0.204 (0.048 - 0.876)	0.032	0.430 (0.117 - 1.585)	0.205
contact health professional before travelling to health facility	0.406 (0.142 - 1.160)	0.092	0.324 (0.114 - 0.919)	0.034	0.212 (0.067 - 0.671)	0.008
do not stop breastfeeding	0.127 (0.040 - 0.404)	0.000	0.078 (0.024 - 0.254)	0.000	0.273 (0.090 - 0.830)	0.022
eating pork is safe	0.283 (0.095 - 0.848)	0.024	0.630 (0.223 - 1.781)	0.384	0.907 (0.301 - 2.736)	0.863
no travelling restriction related to pandemic	0.228 (0.073 - 0.710)	0.011	0.218 (0.074 - 0.638)	0.005	0.282 (0.092 - 0.865)	0.027

^a^OR=odds ratio, ^b^CI=confidence interval

Information about resting and drinking fluids when sick, breastfeeding despite illness, and absence of travel restrictions were significantly less frequent in Australia, Canada, and USA compared with UK (Table 2). Information on hand cleaning, not wearing a mask if not sick, staying home when sick, disposing tissues after use, and cleaning hands after coughing or sneezing were less frequent in Australia compared with UK. Information on sneezing in sleeve or elbow crook was more frequently available in Canada, whereas the recommendation on contacting health professionals before travelling to health facilities was less frequently found in Canada compared with UK (Table 2). Finally, information on wearing a mask when sick if in contact with other people was more frequently found in US compared with UK (Table 2).

The effect of type of website, according to the univariate analysis, showed that pages from Public Health Agencies hosted significantly more information on all items but avoiding touching mouth and nose, improving airflow in living place, practicing good health habits, wearing a mask if sick and in close contact with other people, coughing or sneezing in sleeve or elbow crook, and safety of pork meat.

When adjusting for country and search engine, and taking as reference websites from Public Health Agencies, we found that pages from Universities or Hospitals had a significantly lower probability of reporting recommendations on hand cleaning and staying home when sick (Table 3). Websites from news providers reported less information compared with websites from Health Agencies on all items but improving airflow in the living place, practicing good health habits, wearing a mask when sick and in close contact with other people, informing family and friends about illness, and contacting health professionals before travelling to health facilities. Web pages from drug manufacturers and other commercial agencies less frequently reported information on hand cleaning, reducing time spent in crowds, not needing a mask if not sick, staying home when sick, avoiding contacts with other people, using tissues, disposing tissues and cleaning hands after coughing or sneezing. On the other hand information on improving airflow in the living place was more frequently reported in these web pages compared with those from Public Health Agencies. Pages from other categories, compared with pages from Health Agencies, also less frequently reported information on hand cleaning, staying home when sick, coughing or sneezing in sleeve or elbow crook, not needing to stop breastfeeding, and safety of pork meat; on the other hand information on improving airflow in the living place was more frequently reported in websites from these categories than in pages from Public Health Agencies.

**Table 3:** Adjusted ORs and 95% CI of finding single recommendations during web search by type of website (multivariable analysis, reference type: Health Agencies)

Prevention measures	University Hospital Research		News		Drug industry Commercial		Others	
				
	**OR**^a^ (95% CI^b^)	p	**OR**^a^ (95% CI^b^)	p	**OR**^a^ (95% CI^b^)	p	**OR**^a^ (95% CI^b^)	p
avoid touching mouth and nose	0.348 (0.039 - 3.076)	0.342	0.358 (0.151 - 0.850)	0.020	0.191 (0.033 - 1.087)	0.062	1.509 (0.380 - 5.987)	0.558
hand cleaning	0.019 (0.001 - 0.370)	0.009	0.023 (0.003 - 0.206)	0.001	0.007 (0.000 - 0.108)	0.000	0.080 (0.007 - 0.921)	0.043
avoid close contact with sick people	0.389 (0.045 - 3.388)	0.393	0.291 (0.126 - 0.670)	0.004	0.201 (0.036 - 1.112)	0.066	0.945 (0.274 - 3.261)	0.928
reduce time spent in crowds	0.210 (0.024 - 1.846)	0.159	0.158 (0.064 - 0.395)	0.000	0.161 (0.029 - 0.899)	0.037	0.436 (0.124 - 1.527)	0.194
improve airflow in living place	-	-	1.775 (0.618 - 5.095)	0.286	7.187 (1.187 - 43.519)	0.032	5.391 (1.412 - 20.588)	0.014
practice good health habits	-	-	0.811 (0.341 - 1.931)	0.637	1.859 (0.330 - 10.481)	0.482	2.385 (0.735 - 7.737)	0.148
if you are not sick: mask not needed	0.312 (0.035 - 2.764)	0.295	0.151 (0.060 - 0.383)	0.000	0.167 (0.029 - 0.962)	0.045	0.507 (0.147 - 1.751)	0.282
caregivers: mask when in close contact	0.392 (0.045 - 3.401)	0.395	0.194 (0.084 - 0.450)	0.000	0.351 (0.067 - 1.838)	0.215	0.675 (0.209 - 2.176)	0.510
sick: mask when close contact/travel	0.701 (0.084 - 5.853)	0.743	0.725 (0.331 - 1.587)	0.421	0.657 (0.125 - 3.443)	0.619	1.004 (0.329 - 3.059)	0.994
stay at home	0.017 (0.001 - 0.370)	0.009	0.017 (0.002 - 0.158)	0.000	0.007 (0.000 - 0.105)	0.000	0.079 (0.007 - 0.932)	0.044
take rest and drink fluids	-	-	0.350 (0.152 - 0.803)	0.013	0.434 (0.077 - 2.457)	0.345	0.441 (0.142 - 1.373)	0.158
avoid contact with other people	0.147 (0.016 - 1.387)	0.094	0.093 (0.033 - 0.260)	0.000	0.088 (0.014 - 0.551)	0.009	0.295 (0.076 - 1.140)	0.077
use tissue when coughing-sneezing	0.122 (0.011 - 1.308)	0.082	0.106 (0.031 - 0.359)	0.000	0.046 (0.006 - 0.333)	0.002	0.530 (0.104 - 2.700)	0.444
dispose your tissue after using it	0.090 (0.008 - 1.040)	0.054	0.076 (0.019 - 0.295)	0.000	0.033 (0.004 - 0.260)	0.001	0.392 (0.069 - 2.215)	0.289
sleeve - crook of elbow	-	-	0.360 (0.152 - 0.852)	0.020	0.784 (0.140 - 4.392)	0.782	0.273 (0.079 - 0.948)	0.041
clean your hands after sneezing	0.138 (0.014 - 1.341)	0.088	0.108 (0.037 - 0.309)	0.000	0.084 (0.013 - 0.533)	0.009	0.283 (0.071 - 1.118)	0.072
inform family and friend about your illness	-	-	0.443 (0.159 - 1.239)	0.121	3.207 (0.543 - 18.946)	0.198	0.191 (0.021 - 1.746)	0.143
contact before travelling to health facility	3.063 (0.337 - 27.853)	0.320	0.448 (0.193 - 1.037)	0.061	0.965 (0.173 - 5.375)	0.967	-	-
do not stop breastfeeding	-	-	0.256 (0.106 - 0.620)	0.003	0.458 (0.075 - 2.796)	0.398	0.141 (0.036 - 0.554)	0.005
eating pork is safe	0.403 (0.046 - 3.513)	0.411	0.343 (0.143 - 0.822)	0.016	0.202 (0.035 - 1.152)	0.072	0.293 (0.089 - 0.961)	0.043
no travelling restriction related to pandemic	-	-	0.200 (0.084 - 0.478)	0.000	0.345 (0.062 - 1.931)	0.226	0.350 (0.109 - 1.123)	0.078

^a^OR=odds ratio, ^b^CI=confidence interval

The univariate analysis by search engine showed that websites resulting from Google and Yahoo searches, compared with those resulting from MSN Bing, more frequently reported information on avoiding touching mouth and nose, practicing good health habits, staying home when sick, avoiding contact with other people when sick, respiratory etiquette, informing family and friends when sick, safety of pork meat, and absence of travelling restrictions.

Adjusting for country and type of website, and taking Google as reference, the multivariate analysis (Table 4) showed that while there was no significant difference in the probability of finding the WHO recommendations between Yahoo and Google, websites obtained with MSN Bing less frequently reported information on almost all items, except for hand cleaning, avoiding contact with people and crowds, information on mask wearing, resting and taking fluids, and breastfeeding.

**Table 4:** Adjusted ORs and 95% CI of finding single recommendations during the web search by search engine (multivariable analysis, reference search engine: Google)

Prevention measures	MSN-Bing		Yahoo	
		
	**OR**^a^ (95% CI^b^)	p	**OR**^a^ (95% CI^b^)	p
avoid touching mouth and nose	0.375 (0.148 - 0.954)	0.039	1.405 (0.465 - 4.248)	0.547
hand cleaning	0.318 (0.091 - 1.114)	0.073	2.024 (0.434 - 9.429)	0.369
avoid close contact with sick people	0.829 (0.343 - 2.000)	0.676	1.767 (0.635 - 4.914)	0.275
reduce time spent in crowds	0.558 (0.224 - 1.389)	0.210	1.706 (0.596 - 4.885)	0.320
improve airflow in living place	0.212 (0.070 - 0.640)	0.006	0.580 (0.200 - 1.687)	0.318
practice good health habits	0.273 (0.104 - 0.717)	0.008	1.053 (0.415 - 2.669)	0.914
if you are not sick: mask not needed	0.402 (0.157 - 1.027)	0.057	1.175 (0.406 - 3.402)	0.767
caregivers: mask when in close contact	0.542 (0.221 - 1.331)	0.182	0.966 (0.357 - 2.616)	0.946
sick: mask when close contact/travel	0.594 (0.255 - 1.380)	0.226	1.220 (0.480 - 3.106)	0.676
stay at home	0.167 (0.043 - 0.643)	0.009	1.484 (0.307 - 7.188)	0.624
take rest and drink fluids	0.590 (0.242 - 1.440)	0.247	1.175 (0.447 - 3.090)	0.743
avoid contact with other people	0.358 (0.133 - 0.966)	0.043	1.610 (0.517 - 5.010)	0.411
use tissue when coughing-sneezing	0.270 (0.087 - 0.843)	0.024	1.139 (0.299 - 4.334)	0.848
dispose your tissue after using it	0.285 (0.089 - 0.915)	0.035	1.167 (0.300 - 4.539)	0.823
sleeve - crook of elbow	0.304 (0.118 - 0.784)	0.014	0.997 (0.382 - 2.602)	0.995
clean your hands after sneezing	0.201 (0.060 - 0.669)	0.009	0.528 (0.172 - 1.624)	0.265
inform family and friend about your illness	0.201 (0.060 - 0.669)	0.009	0.528 (0.172 - 1.624)	0.265
contact health professional before travelling to health facility	0.365 (0.140 - 0.949)	0.039	0.907 (0.341 - 2.412)	0.845
do not stop breastfeeding	0.592 (0.225 - 1.559)	0.289	0.894 (0.318 - 2.515)	0.832
eating pork is safe	0.383 (0.158 - 0.927)	0.033	1.709 (0.595 - 4.905)	0.319
no travelling restriction related to pandemic	0.256 (0.099 - 0.663)	0.005	0.432 (0.156 - 1.201)	0.108

^a^OR=odds ratio, ^b^CI=confidence interval

## Discussion

Our study shows an overall high probability of finding on the web appropriate information regarding hand washing and main steps of the respiratory etiquette consistent with WHO recommendations, whereas other recommendations regarding behaviors that can prevent human-to-human transmission of influenza A(H1N1)v are rarely found during a generic web search on pandemic flu. Moreover, the probability of finding appropriate information for H1N1 influenza prevention is affected by country, type of website, and web search engine.

General information on respiratory etiquette consistent with WHO recommendations is rarely complete and reported, especially when considering information on covering mouth and nose with sleeve or elbow crook when coughing or sneezing.

Recommendations about practicing good health habits, improving airflow in living place, informing family and friends when sick, as well as those about breastfeeding (continue breastfeeding despite illness) and contacting health professionals before travelling to health facilities are also rarely shown, and would deserve better implementation.

In the last years access to the Internet and use of the World Wide Web have been constantly increasing. Users often seek health information on the web [4] and the Internet has played a central role as an information source on H1N1 influenza, as documented by the volume of searches launched on Google on this subject [8].

Therefore, giving on the web appropriate advices on prevention to users typically surfing the web for information on pandemic flu should be a privileged strategy for information dissemination. Thus, it is crucial to measure the probability that an average Internet user has to access appropriate information.

In our study we found a higher availability of information on H1N1 influenza for most recommendations in searches performed in the UK compared with the other countries.

Cultural models, local habits, different web dissemination strategies, and different moments in the evolution of the pandemic may explain some of these differences. Moreover, certain specific recommendations were more frequent in some countries, as documented by the higher probability of finding information on sneezing in the sleeve or elbow crook in Canada, and wearing a mask when sick in the US compared with the UK. On the other hand, a lower coverage of WHO recommendations in Australia might be associated with the observation that in this country the pandemic peak was already over when this study was conducted.

We observed that websites obtained through MSN-Bing show less information about pandemic flu prevention compared to websites obtained through Google or Yahoo.

Finally, we found that the majority of the websites were from news providers, followed by pages from Public Health Agencies. However, news websites provide significantly less information regarding preventive measures compared to Public Health Agencies. The same happens for commercial websites and drug companies websites.

The major Internet search engines use complex algorithms to rank search results. Websites from major organizations tend to appear first when a search is carried out, and the risk of exploring sites that provide inaccurate advice should be small [9]. Our search shows that usually the first search hit, in the large majority of cases, is a website from a Public Health Agency. Nevertheless, news providers, often found in the first page of search results, in most cases do not provide adequate advice.

Therefore, taking into account the higher rank that news providers get on the major search engines in a generic search on swine flu, this kind of websites should improve reporting of complete information on influenza prevention, or provide links to websites showing comprehensive sets of adequate recommendations. This could be an effective strategy for a broader dissemination of appropriate influenza prevention behaviors.

The main limitation of our study is that the analysis was performed only once, while information found on the web is constantly changing. Since the pandemic virus started circulating, knowledge on its epidemiological characteristics has improved, and interest towards this subject may have changed. We cannot say if the availability of recommendations on prevention has changed over time. We did not consider the advertised links that appear on the side and on the top of Google search result page, which lead to commercial sites. Another limitation is that our analysis was performed only on English websites from English-speaking countries, and may therefore not be generalized to other countries or to websites in other languages. Although further studies are needed to address this issue, we think that a study of this kind can be useful to assess web availability of recommendations on prevention in specific contexts, and can therefore help improving strategies of public health information delivery to the general public.

## Conclusions

The Internet is a rich and efficient source of information about infectious diseases and their prevention. Its role in social marketing can be crucial. Integrating its potentials with those of other media can guarantee a broad spread of information. Moreover, public health interventions transmitted through this medium could target groups with peculiar characteristics [6].

In conclusion, in order to guarantee an adequate use of this instrument by the public, health professionals, who are still one of the main sources of information regarding health matters, should guide patients to other reliable sources (information prescription [9,10]) at the same time, deeper attention should be paid by Public Health Agencies towards quality of information and search engine optimization; studies like the one presented in this article should be used more often to improve delivery of public health information to the general public.

## Competing interests

The authors declare that they have no competing interests.

## Authors' contributions

FG coordinated the study, participated in the webpages review process and in the writing process. MR participated in the design and in the maintenance of the database. EP participated in the webpages review process. CR helped to draft the manuscript. LR performed the statistical analysis. DL helped in the webpages review process. AET conceived the study, participated in its design and coordination and helped to draft the manuscript. All authors read and approved the final manuscript.

## Pre-publication history

The pre-publication history for this paper can be accessed here:

http://www.biomedcentral.com/1471-2458/10/561/prepub

## Supplementary Material

Additional file 1 **URL list**. List of the URLs resulting from the searches launched on August 13^th ^2009 from the 4 proxy servers (Australia, Canada, UK, USA) on the 3 search engines (Google, Yahoo, MSN-Bing). Click here for file
